# Structural insights into assembly of human mitochondrial translocase TOM complex

**DOI:** 10.1038/s41421-021-00252-7

**Published:** 2021-04-13

**Authors:** Zeyuan Guan, Ling Yan, Qiang Wang, Liangbo Qi, Sixing Hong, Zhou Gong, Chuangye Yan, Ping Yin

**Affiliations:** 1 National Key Laboratory of Crop Genetic Improvement and National Centre of Plant Gene Research, Huazhong Agricultural University, Wuhan, Hubei 430070 China; 2 Laboratory of Magnetic Resonance and Atomic Molecular Physics, Wuhan Institute of Physics and Mathematics of the Chinese Academy of Sciences, Wuhan, Hubei 430071 China; 3 Beijing Advanced Innovation Center for Structural Biology, Tsinghua-Peking Joint Center for Life Sciences, School of Life Sciences, Tsinghua University, Beijing, 100084 China

**Keywords:** Protein translocation, Cryoelectron microscopy

Dear Editor,

Mitochondria contain ~1000–1500 proteins, 99% of which are encoded by nuclear genes and correctly transported into the mitochondrial subcompartment after translation in the cytoplasm^1^. The TOM complex is the main entry gate for the transfer of protein precursors from the cytosol to other mitochondrial translocase complexes. The TOM holo-complex consists of the channel-forming β-barrel protein Tom40 and six other subunits, including the receptor proteins Tom20, Tom22, and Tom70 and the regulatory Tom proteins Tom5, Tom6, and Tom7; each subunit contains a single α-helical transmembrane (TM) segment. In the absence of Tom70 and Tom20, the remaining five components form a stable TOM core complex^2,3^. The process through which α-helical and β-barrel membrane proteins assemble into a functional TOM complex remains to be further studied.

In yeast, the TOM complex has been characterized as two or three pores via electron microscopy (EM) and crosslinking analyses, and these correspond to the dimeric and trimeric TOM complexes, respectively^2,4–7^. The TOM complex can exhibit dynamic conversion between dimer and trimer forms^7^, suggesting that the TOM complex undergoes structural rearrangement during various stages of preprotein translocation across the outer membrane. Most recently, high-resolution cryo-EM structures of dimeric TOM core complexes from *Saccharomyces cerevisiae* have been reported^8,9^, and the TOM tetramer, which is equivalent to a dimer of dimers, has also been observed^8^. However, little structural information is available for the trimeric TOM complex.

More importantly, in humans, the TOM complex is related to some mitochondrial diseases, such as Parkinson’s disease (PD). In damaged mitochondria, the TOM complex arrests PINK1, which then recruits and activates Parkin to induce mitophagy^10^. The failure to remove dysfunctional mitochondria might lead to early-onset PD development. Elucidation of the assembly of dimeric and trimeric human TOM complexes could not only provide insights into the mechanism of protein translocation via the TOM complex but also lay a solid foundation for the development of therapies for mitochondrial diseases.

Here, we present the structures of the human dimeric TOM core complex at 3.0 Å resolution and the trimer conformation at 4.3 Å resolution. First, we co-expressed seven components of the TOM complex in the human embryonic kidney (HEK) 293F cells. The only fusion of the Flag tag to the C-terminus of Tom22 gave rise to a stable protein complex. After Flag-tag affinity purification followed by gel filtration, the resultant TOM complex displayed good quality. All seven components were clearly monitored by SDS-PAGE (Fig. 1a). The peak fractions, which migrated to a single band of ~400 kDa on blue native PAGE (BN-PAGE), were pooled for cryo-sample preparation (Supplementary Fig. S1). After the initial 2D classification, 3D classification and refinement of the cryo-EM particle images yielded a final 3D EM reconstruction map with an overall 3.0 Å resolution (Fig. 1b; Supplementary Figs. S2 and S3, Table S1). The density map is of sufficient quality to allow us to build Tom40, Tom22, Tom5, Tom6, and Tom7 but is not adequate for tracing Tom20 and Tom70. This suggested that Tom20 and Tom70 were dissociated from the complex under cryo-EM sample preparation, in line with the results of previous studies showing that these two receptors are dynamically associated with the core complex with lower affinity. Most regions exhibit excellent density and contain aromatic and bulky residues that are easily recognizable for model building (details in Supplementary Table S2).

**Fig. 1:**
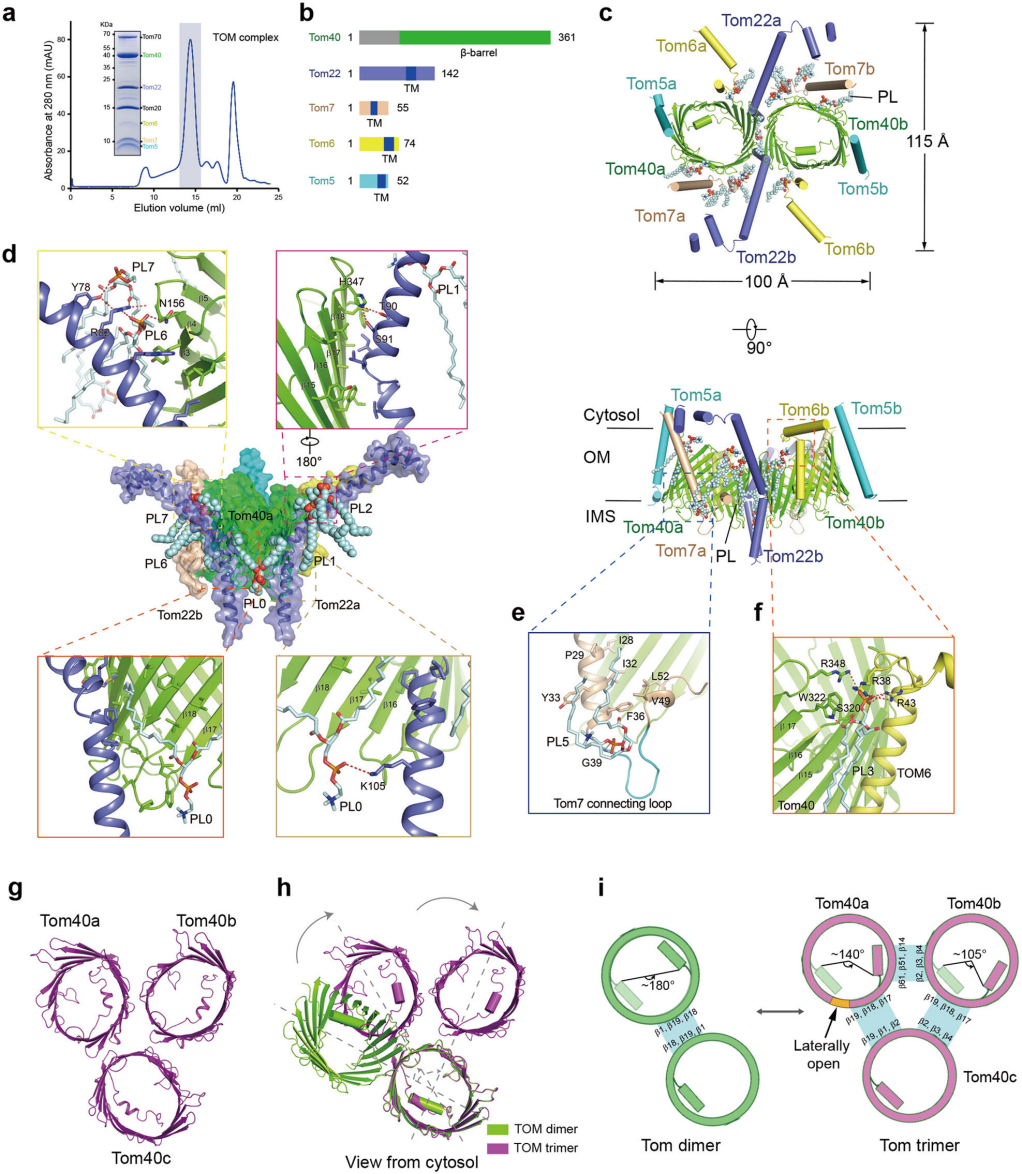
Cryo-EM structures of the human dimeric TOM core complex and trimeric TOM. **a** Representative gel filtration chromatography profile of the purified TOM core complex. **b** Schematic diagram of each subunit of the TOM core complex. **c** Top view of the overall structure of the TOM core complex (top panel) and side view (bottom panel) with each subunit individually colored. Lipids are indicated with spheres. OM indicates the outer membrane. IMS represents intermembrane space. **d** Interactions between Tom40 and Tom22 with lipid molecules as bridges. Tom40 and Tom22 are colored in chartreuse and blue, respectively. The phosphate group of PL0 forms a hydrogen bond with K105 of Tom22, and the acyl chains of PL0 hydrophobically interact with Tom40 and Tom22. PL6 interacts with N156 of Tom40 and R82 of Tom22, and PL7 interacts with Y78 and R82 of Tom22. **e** Interactions between Tom7 and the PL5 molecule. The connecting loop in Tom7 is labeled. **f** PL3 is involved in the interaction between Tom40 and Tom6. PL3 interacts with R38 and R43 of Tom6 and three residues (S320, W322, and R348) of Tom40. **g** Overall structure of the trimeric TOM complex from the cytosol view. Three Tom40s were named Tom40a, Tom40b, and Tom40c. **h** Structural alignment between the Tom40 dimer (chartreuse) and Tom40 trimer (purple) showing the rotation of Tom40. **i** Schematic diagram of the dimer-trimer conversion model.

The overall structure of the TOM core complex contains two copies of Tom40, Tom22, and small Tom proteins, which form two symmetrical pores. The height and width of the structure are ~100 Å and 115 Å, respectively (Fig. 1c). Two Tom40s, as elliptical barrels, were tilted and located close to each other. Two Tom22s were embedded in the symmetry plane of the entire structure to bridge two Tom40s. Small Tom proteins closely surrounded Tom40. Based on the cryo-EM map, 11 lipid-like EM densities were found to encircle Tom40. We assigned these phospholipids (PL) as phosphatidylcholine (PC) in the structure and numbered these lipids from PL0 to PL10 according to their relative position (Supplementary Fig. S4a, b). These lipids participated in the interactions of Tom6, Tom7, or Tom22 with Tom40 and thus strengthened the α helix-β sheet contacts (details are described below).

Tom40 contained 361 amino acids; the N-terminal extended helix (residues 76–99) inside the barrel and the following 19 β-strands (residues 100–361) were fully resolved, whereas the N-terminal region (residues 1–75) failed to be resolved. The N-terminal extended helix interacted with β9–β19 on one side and occupied almost half of the pore, suggesting a channel-gating role for this helix. β1 and β19 were arranged in a parallel manner with five hydrogen bonds between them, whereas the remaining β-strands were antiparallel (Supplementary Fig. S4c, d). The Tom40 β-barrel might open laterally through β1 and β19 to allow precursor release into the outer membrane^11^. In addition, β1 and β19 were hidden in the dimer interface (Fig. 1c), and β19 was buttressed by Tom22 (Fig. 1d, top right panel), making its opening difficult. We speculated that β1 and β19 might undergo notable conformational changes due to β-barrel pore rotation at different oligomeric states, resulting in their exposure and ultimately in substrate release. Tom40 from higher eukaryotes has extra N-terminal segments exposed to the cytosol^12^. An additional unassigned EM density in the micelle located between β7–β9 of Tom40 and Tom5 was near the N-terminus of Tom40 (Supplementary Fig. S5). Although it appeared to be part of Tom40, the lack of obvious density at the N-terminus extension likely reflects their flexible nature and potential functions.

Tom22 comprises an N-terminal cytosolic domain, which is critical for binding to preproteins, a TM helix, and a small IMS portion^13^. In the structure, residues 25–52 (part of a cytosolic domain) of Tom22, which contain a glutamic acid/aspartic acid-rich segment (residues 29–42), were partially resolved and built as polyalanine. It was negatively charged enriched and exhibited an extended conformation, which might act as a platform for preprotein retention (Supplementary Fig. S6a, b). Next to this segment, a long helix (residues 60–114) bent an angle of ~50° at P96 and passed through the membrane. The cytosolic part (residues 65–82) consisting of hydrophilic amino acids on one side and hydrophobic amino acids on the other side was possibly related to binding to the amphiphilic presequences of preproteins via hydrophobic interactions (Supplementary Fig. S6c).

Five lipids (PL0–2, PL6, and PL7) were involved in the assembly of Tom22 and Tom40 (Fig. 1c, middle panel). The unique lipid PL0 sitting at the symmetry axis of the dimer with its head groups facing the IMS sealed the cavity formed by two copies of Tom22 and Tom40 (Fig. 1c, bottom panel). PL1 (PL6) and PL2 (PL7) were located between the cytosolic part of the Tom22 helix and Tom40 β3–β5 (Fig. 1c, top left panel). Notably, the closeness of the head groups of these two lipids made it possible to build cardiolipin (CL) at the coordinates of PL1 and PL2 (Supplementary Fig. S6d).

Tom5, Tom6, and Tom7 were located at β9–β11, β13–β15, and β1–β8 of Tom40, respectively. The TM helices of them were attached to Tom40 mainly via hydrophobic interactions, and these helices also formed several hydrogen bonds with Tom40 (Supplementary Fig. S7a, b). Tom6 contained two helices and exhibited an inverted L shape. The α1 of Tom6 in the cytosol presented an extended conformation and appeared to be able to contact Tom22, and this finding was in accordance with previous studies, which showed that Tom6 was important for stimulating the assembly of Tom22 with Tom40^7,9^ (Supplementary Fig. S7c). Tom7 consisted of an N-terminal long helix inclined to Tom40, a connecting loop exposed to IMS (Fig. 1d), and a C-terminal helix extended into the membrane; it interacted widely with Tom40 (Supplementary Fig. S7d, e). Lipids (PL3 and PL4) were observed near Tom6 and Tom7 and served as a bridge for these proteins with Tom40 (Fig. 1e; Supplementary Fig. S7d). Furthermore, PL5 was encircled by two helices of Tom7. The head groups of PL5 fixed the connecting loop of Tom7, resulting in the exposure of this loop to IMS. One acyl chain stabilized two helices via hydrophobic interactions (Fig. 1d).

Our structure showed that the β-barrel protein Tom40 and four helical TM proteins were directly organized together through interactions. During this process, lipids acted as a bridge to reinforce the α-β assembly. To corroborate this observation, we designed molecular dynamics (MD) simulations. Because the type of lipid at a specific position was unascertained, we adopted similar PL, such as PC, phosphatidylethanolamine (PE), and phosphatidylserine (PS), for the MD simulations. In the absence of these lipids, the entire structure becomes more dynamic (Supplementary Fig. S8a). In contrast, lipids had little effect on the stability of Tom40 because Tom40 was already a relatively stable β-barrel protein (Supplementary Fig. S8b). Intriguingly, the structure of Tom6 became more dynamic in the presence of PL3, which suggested that lipids might play a role in mediating Tom6 assembly or substrate binding (Supplementary Fig. S8c, d).

After superposition of the human TOM core complex with the yeast TOM core complex (PDB: 6JNF), notable differences were observed in the C terminus of Tom40, the TM helix angle of Tom22, and the extension orientation of the cytoplasmic region of Tom6 (Supplementary Fig. S9a, b). In yeast, Tom40 has a 14-amino acid negatively charged C-terminal tail that follows the last β-strand and extends to the IMS. Deletion of the C-terminal segment of Tom40 (Δ364–387) impaired the accumulation of the presequence-containing Oxa1 precursor at the TOM complex^9^. In contrast, the C-terminus of hTom40 terminated at the last β-strand (G361) without a tail, which suggested that other components in the TOM complex might function like the C-terminal tail in yTom40 (Supplementary Fig. S4d). The connecting loop of Tom7 was observed on the periphery of the Tom40 C-terminus. Compared with that of yTom7, the connecting loop of hTom7 was a negatively charged patch and might be involved in precursor protein binding on the IMS side (Supplementary Fig. S10a, b).

As a major entry gate, the TOM complex could act as both a pore and a molecular machine for lateral protein release into the outer membrane^11,14^. In the structure, Tom6 formed a region with a negatively charged surface, concurrently close to the precursor binding region of Tom22 and the negatively charged patch of Tom40. We speculated that the precursor protein might slide into the pore of Tom40 through these three negatively charged regions enriched in the presequence pathway (Supplementary Fig. S11a, b). In the dimeric TOM core complex, β1 and β19 in the Tom40 barrel were hard to open laterally. However, in the trimeric TOM complex, rotation of the barrel to expose β1 and β19 might occur.

Previous studies have proposed that the TOM holo-complex might be three pores or a trimer^6,7^. To obtain the cryo-EM structure of the trimeric TOM complex, we constructed both Tom70 and Tom22 with Flag tags to improve the protein quantity of Tom70. A stable band of ~720 kDa was observed on BN-PAGE, and this band was abundant in Tom20, Tom22, Tom40, and Tom70. The cryo-EM examination revealed particles with six pore features in the 2D classification (Supplementary Fig. S12). After 3D reconstitution, we obtained a density map with an overall resolution of 6.3 Å for C1 symmetry and a density map with an overall resolution of 4.3 Å after applying C2 symmetry (Supplementary Fig. S12). Only Tom40 could be modeled, but the densities with helical features were inadequate for modeling. Accordingly, we constructed a model based on the characteristics of the N-terminal helix of Tom40 (Supplementary Fig. S13e, f) and ultimately modeled a hexameric TOM that consists of two trimeric Tom40 (Fig. 1f).

By comparing the Tom40 dimer and trimer structures, three pores were found to spin and revolve around an axis to a certain degree (Fig. 1g). Using one Tom40 as a reference in dimeric TOM, the other Tom40 protomers exhibited a 180° rotation. The contact interface between the two Tom40s was β1–β19–β18. In contrast, the rotation degrees in the trimeric TOM were ~140° and 105° (Fig. 1h). Thus, the Tom40 trimer generated three interfaces, namely, interface I between Tom40a (β19–β18–β17) and Tom40c (β19–β1–β2), interface II between Tom40a (β14–β15–β16) and Tom40b (β4–β3–β2), and interface III between Tom40b (β19–β18–β17) and Tom40c (β2–β3–β4). Interface I is similar to that in the dimeric TOM complex. This conformational change exposes β1 and β19 in Tom40a to the membrane, which makes it possible for Tom40a to open laterally.

To investigate the existence of the TOM trimer in human mitochondria, we performed *in* *organelle* cross-linking assay, which was applied to characterize yeast TOM trimer^7^. First, we generated a cysteine-free Tom40 variant to avoid nonspecific cross-linking. Based on the structures, we then introduced Cys at the dimer interface (L339), trimer interface (II: Y129/S320, V115/V286, V105/F301; III: L339/V133, L339/F131), or remote from interfaces (S183). As expected, a cross-linked product (~90 kDa) was evident for Tom40^L339C^ but invisible for Tom40^S183C^. The introduction of Cys at interface II or III yielded a cross-linked band of ~130 kDa, which is indicative of trimeric Tom40. Collectively, these results are consistent with the structural observation of the trimeric TOM complex.

In summary, our data reveal the high-resolution structure of human dimeric TOM core complexes and the near-atomic-resolution structure of the human trimeric TOM complex. A 3.4-Å-resolution cryo-EM structure of dimeric human TOM core complex was reported in *Cell Discovery* during the manuscript revision process^15^. These findings provide a structural basis for understanding the molecular transport mechanism of the human TOM complex. We elucidated how lipids link the α-helix and β-sheet components. Moreover, we provided biochemical evidence of the trimeric TOM complex in vivo, which suggests that the human TOM complex can exist in different oligomer states in a cell to facilitate substrate transport. For instance, in depolarized mitochondria, trimeric TOM might laterally release PINK1-like substrates into the outer mitochondrial membrane (OMM) and lead to accumulation in the OMM^10^. However, obtaining a high-quality cryo-EM map of the trimeric TOM complex to explain the roles of other TOM components in trimer assembly remains an unprecedented challenge. Furthermore, obtaining the elaborate structures of the TOM complex with the precursor is of great importance for fully elucidating the mitochondrial protein translocation mechanism.

## Supplementary information

supplementary information
