# Supplementary material for: Structural insights into assembly of human mitochondrial translocase TOM complex
Source: Cell Discov. 2021 Apr 13;7:22. doi: 10.1038/s41421-021-00252-7 (PMC8041818; doi:10.1038/s41421-021-00252-7)
Supplement: Supplementary file 1 — supplementary information [file 41421_2021_252_MOESM1_ESM.pdf]

Structural insights into assembly of Human Mitochondrial Translocase TOM Complex

Zeyuan Guan[1*], Ling Yan[1*], Qiang Wang[1], Liangbo Qi[1], Sixing Hong[1], Zhou Gong[2], Chuangye Yan[3], and Ping Yin[1]

[1]National Key Laboratory of Crop Genetic Improvement and National Centre of Plant Gene Research, Huazhong Agricultural University, Wuhan 430070, China.

[2] Laboratory of Magnetic Resonance and Atomic Molecular Physics, Wuhan Institute of Physics and Mathematics of the Chinese Academy of Sciences, Wuhan 430071, China.

[3]Beijing Advanced Innovation Center for Structural Biology, Tsinghua-Peking Joint Center for Life Sciences, School of Life Sciences, Tsinghua University, Beijing 100084, China.

Correspondence: Ping Yin (yinping@mail.hzau.edu.cn) or Chuangye Yan (yancy2019@mail.tsinghua.edu.cn)

*These authors contributed equally to this work.

Supplementary information

Material and Methods

Transient expression of the human TOM complex

The codon-optimized full-length cDNAs of the subunits of the human TOM complex (Uniprot number, Tom40: O96008; Tom22: Q9NS69; Tom5: Q8N4H5; Tom6: Q96B49; Tom7: Q9P0U1; Tom20: Q15388, Tom70: O94826) were synthesized by General Biosystems Company. The seven cDNAs were sequentially cloned into the pMlink vector[1]. Tom22 contains a C-terminal 3×Flag tag. For dimer of trimeric TOM complex, Tom22 and Tom70 both contain a C-terminal 3×Flag tag. To increase the co-expression efficiency of multiple subunits, Tom40, Tom22, and Tom20 were reconstituted into a single plasmid A, and the three small Toms (Tom5, Tom6 and Tom7) were reconstituted into another single plasmid B. Expi293F™ (Invitrogen) cells were cultured in SMM 293TI medium (Sino Biological Inc.) at 37°C under 5% CO_2 in a ZCZY-CS8 shaker (Shanghai Zhichu Instrument co., Ltd.). The cells were diluted into 2.0×10^6 cells ml^{-1} with fresh medium for further transfection when the cell density reached 3.5×10^6 ~ 4.0×10^6 cells ml^{-1}. For 1 liter cell culture, a total 2 mg plasmids (plasmid A: plasmid B : pMlink-Tom70 = 1:1:1) were pre-incubated with 4 mg linear polyethylenimines (PEIs) (Polysciences) in 50 ml fresh medium for 20 min followed by adding the mixture into the diluted cells. The transfected cells were cultured for 48 hours before harvest.

Mitochondria Preparation

The cells overexpressing the TOM complex or dimer of trimeric TOM complex were harvested by centrifugation at 800 g for 20 min and washed by cold PBS, then resuspended in the buffer A containing 10 mM Tris-HCl (pH 7.5), 70 mM sucrose, 210 mM mannitol, 1 mM EDTA, 1mg ml^{-1} BSA, and 1mM PMSF. The cells were disrupted using Dounce homogenizer (Sigma) for 65 cycles on ice. The obtained homogenate was centrifuged at 3,000 g for 10 min. The supernatant was further centrifuged at 20,000 g for 20 min to obtain the crude mitochondrial pellets. The pellets were resuspended in buffer B (10 mM Tris pH 7.5, 250 mM sucrose, 60 mM KCl and 0.1 mM EDTA) and further purified by density gradient centrifugation over a 50%-

30%-18% percoll (GE Healthcare) gradient at 60,000 g at 4°C for 20 min. The clear mitochondria layer was obtained carefully for further protein purification.

Purification of the TOM complex

The TOM complex was extracted from pure mitochondria by 1% digitonin (BIOSYNTH®, cat number: D3203) in lysis buffer containing 25 mM HEPES (pH 7.4), 100 mM KAc, 10 mM Mg Ac_2, 0.1 mM EDTA, 10% glycerol, 1 mM PMSF, 2 μg ml^{-1} pepstatin A, 2 μg ml^{-1} aprotinin, and 2 μg ml^{-1} leupeptin at 4°C for 1.5 h. The extract was centrifuged at 23,000 g for 30 min to remove the unsoluble component. The supernatant was incubated with anti-Flag G1 affinity resin (Genscript) at 4°C for 1 h, and then washed with 30 bed volumes of lysis buffer added with 0.1% digitonin. The protein was eluted with lysis buffer added with 0.1% digitonin and 300 μg ml^{-1} Flag peptide (Genscript). For cryo-EM study, the protein solution was further purified by Superose-6 increase 10/300 column (GE Healthcare) with buffer containing 25 mM HEPES (pH 7.4), 100 mM KAc, 10 mM $MgAc_2$, and 0.06% digitonin[2]. The peak fractions were pooled and concentrated to 10 mg ml^{-1} for further cryo-EM study.

Blue native-PAGE analysis

Blue native PAGE[3] technique was used to determine purified TOM complex. The protein sample was mixed with 10×loading buffer (0.1% w/v Ponceau S, 50% w/v glycerol) and subjected to 4%-16% blue native PAGE mini gel (Invitrogen: BN1002BOX) electrophoresis at 4°C. Cathode buffer A contained 50 mM Tricine, 15 mM Bis-Tris (pH 7.0), and 0.02% Coomassie G-250. Cathode buffer B was similar to cathode buffer A in formula, but 0.02% Coomassie G-250 was removed. Anode buffer contained 50 mM Bis-Tris pH 7.0.

Mass spectrometry analysis

The TOM complex proteins were separated by SDS-PAGE or BN-PAGE, the gel bands of interest were excised from the gel, reduced with 5 mM of dithiotreitol, and alkylated with 11 mM iodoacetamide. In gel digestion was then carried out with sequencing grade modified trypsin in 50 mM ammonium bicarbonate at 37°C overnight. The peptides were extracted twice with 0.1% trifluoroacetic acid in 50% acetonitrile aqueous solution for 30 min. Extracts were

then centrifuged in a speedvac to reduce the volume. Tryptic peptides were redissolved in 20 μl 0.1% TFA and analyzed by LC-MS/MS.

Cryo-EM Data Acquisition

For cryo-EM sample preparation, 3.5 μl aliquots of the recombinant human complex (~ 10 mg ml^{-1}) were dropped onto glow-discharged holey carbon grids (Quantifoil Au R1.2/1.3, 300 mesh), blotted with a Vitrobot Mark IV (ThemoFisher Scientific) for 3.5 seconds with 100% humidity at 8°C, and plunged into liquid ethane cooled by liquid nitrogen. The TOM complex sample was imaged with an FEI Titan Krios transmission electron microscope at 300 kV with a magnification of 29,000 ×. Images were recorded by a Gatan K2 Summit direct electron detector in the counting mode. Defocus values varied from -1.8 to -2.5 μm. Each image was dose fractionated to 32 frames with a total electron dose of 60 e^{-} $Å^{-2}$ and a total exposure time of 8.0 s. SerialEM was used for fully automated data collection[4]. All stacks were motion corrected using MotionCor2 with a binning factor of 1, resulting in a pixel size of 1.014 Å and dose weighting was performed concurrently[5]. The defocus values were estimated using Gctf[6]. Dimer of trimeric TOM complex sample was imaged on FEI Titan Krios transmission electron microscope at 300 kV with a magnification of 105,000 ×. Images were recorded by a Gatan K2 Summit direct electron detector with slit width of 20 eV on the energy filter and a preset defocus range from -1.8 to -2.5 μm. Each image was dose fractionated to 32 frames with a total electron dose of 48 e^{-} $Å^{-2}$ and a total exposure time of 5.6 s. AutoEMation was used for fully automated data collection. All stacks were motion corrected using MotionCor2[5], resulting in a pixel size of 1.091 Å, and dose weighting was performed concurrently. The defocus values were estimated using Gctf[6].

Data processing

A diagram of the procedures for data processing is presented in Supplementary Fig. S2. The first datasets (TOM core complex) had 7,632 micrographs. A total of 6,855 good micrographs were selected, from which 3,094,753 particles were auto-picked using RELION[7]. After reference-free 2D classification, 1,934,386 good particles were selected for 3D classification. Multi-reference 3D classification was performed in RELION[7]. Then, a total of 821,119 particles

were selected from good classes and transferred to the cryoSPARC[8] software package for further processing, followed by several rounds of ab-initio reconstruction, heterogeneous refinement. Particles belonging to the best class were selected followed by non-uniform refinement and local refinement, applying C2 symmetry, yielding a particle density with an estimated resolution of 3.0 Å based on FSC[9].

The second datasets (dimer of trimeric TOM) had 9,838 micrographs, which was directly imported into cryoSPARC[8]. 2D classification resulted in 2,277,464 particles with 2-pore and 6-pore conformations. The particle with 6-pore feature were selected for training template, and Topaz software was used for particle picking. Then, class averages of 6-pore particles were selected and subjected to three-dimensional (3D) classification. A final set of 59,104 particles resulted in a reconstruction of 4.3 Å resolution with imposed C2 symmetry.

Model building and structure refinement.

We combined homology modeling and de-novo model building to generate the atomic models. Identification of Tom40 protein was facilitated by the homology structure from Neurospora crassa (PDB code: 5O8O)[10]. The atomic model of Tom40 were generated by CHAINSAW and the backbone was manually adjusted using COOT[11]. After that, automated model rebuilding was performed with RosettaCM using the adjusted model as the template and the experimental cryo-EM density as a guide[12-14]. Then the hydrogen atoms of the generated model were removed and model building was further performed manually using COOT. The other four components (Tom5, Tom6, Tom7, Tom22) were built de novo into the EM density map in COOT. Due to limited resolution (4~6 Å), only poly-Ala helix was built for the N-terminus of Tom22 (Residues 29-50). Several cryo-EM density lobes resemble phospholipids, but the quality of these densities was insufficient for assignment of the phospholipids. For convenience, we assigned these lipids as phosphatidylcholine (PC), which were in line with the lipid-like EM density map. We numbered these lipids according to their relative position (PL0-PL10). Apart from unique PL0, PL1 and PL6, PL2 and PL7, PL3 and PL8, PL4 and PL9, PL5 and PL10 were symmetric. Subsequently, the models were refined against the corresponding maps by PHENIX[15] in real space (phenix.real_space_refine) with secondary structure restraints.

The final atomic model was validated through examination of the Molprobity scores and

statistics of the Ramachandran plots[16]. The cryo-EM density map for the human dimeric TOM core complex has been deposited in EM Database under the accession code EMD-30421. The corresponding atomic coordinates have been deposited in the Protein Data Bank under accession code 7CP9. The cryo-EM density map for the human dimer of trimeric TOM complex has also been deposited in EM Database under the accession code EMD-30422.

Molecular dynamics simulations

The all-atom molecular dynamic simulations were performed using AMBER16 package[17]. The structures of TOM core complex, Tom6-Tom40, Tom7-Tom40 were used as the initial conformation, in lipid-bound (PC, PE, PS) state or free state (Lipid was removed from the bound conformation). All the simulations were performed in the presence or absence of lipids for all the structures. For Tom40 alone, PL0-PL4 were tested for simulation. For Tom40 and Tom6, PL3 was retained or removed for simulation. For Tom40 and Tom7, simulation was performed with or without PL5. The amber ff14SB force field was constructed for protein. Parameters for different lipids were set using AM1-BCC[18] charge model in general amber force field (GAFF) [19]. The whole system was solvated in TIP3P water box with 10 Å padding in every direction. The Particle Mesh Ewald algorithm was used to treat the long-range electrostatics. The non-bond interaction cutoff was set as 10 Å. The structures were first energy-minimized and equilibrated for 1 ns before the simulation. All simulation procedure lasted 100 ns to produce 1000 snapshots at 100 ps interval. The RMSD analysis was performed using CPPTRAJ module in AMBER16.

Chemical crosslinking

We generated Tom40 constructs using pMlink plasmid with C-twin strep tag encoding the WT form as a template. The cysteines free form of Tom40 (C74S, C76E, C86L, C90V, C228Q) were replaced by PCR. Subsequently, cysteine residues were introduced at specific sites for chemical crosslink experiment. Isolated mitochondria in buffer A were treated with 1 mM M2M (1,2-ethanediyl bismethanethiosulfonate, Toronto Research Chemicals, Canada) for 30 min in room temperature .Then mitochondria were solubilized on ice for 30 min with 1% digitonin in BN-lysis buffer containing 50 mM Tris-HCl (pH 7.4), 150 mM NaCl, 10% glycerol, and 1 mM

PMSF. After centrifuged at 14,000 rpm for 10 min, samples were analysis by nonreducing SDS-PAGE and BN-PAGE. Reactions were quenched by addition of 50 mM DTT.

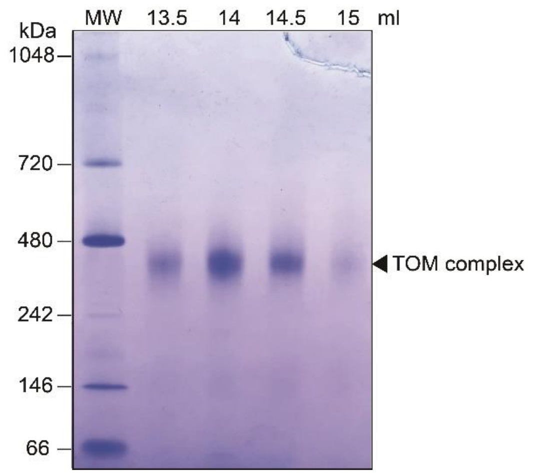

Supplementary Fig. S1 BN-PAGE analysis of the human dimeric TOM core complex. The TOM core complex sample was analyzed by BN-PAGE. A native marker (Thermo Fisher, Cat number: LC0725) was used to reveal the molecular weight of the TOM complex. The eluted volume are corresponded to the gel filtration chromatography in Supplementary Fig 1a.

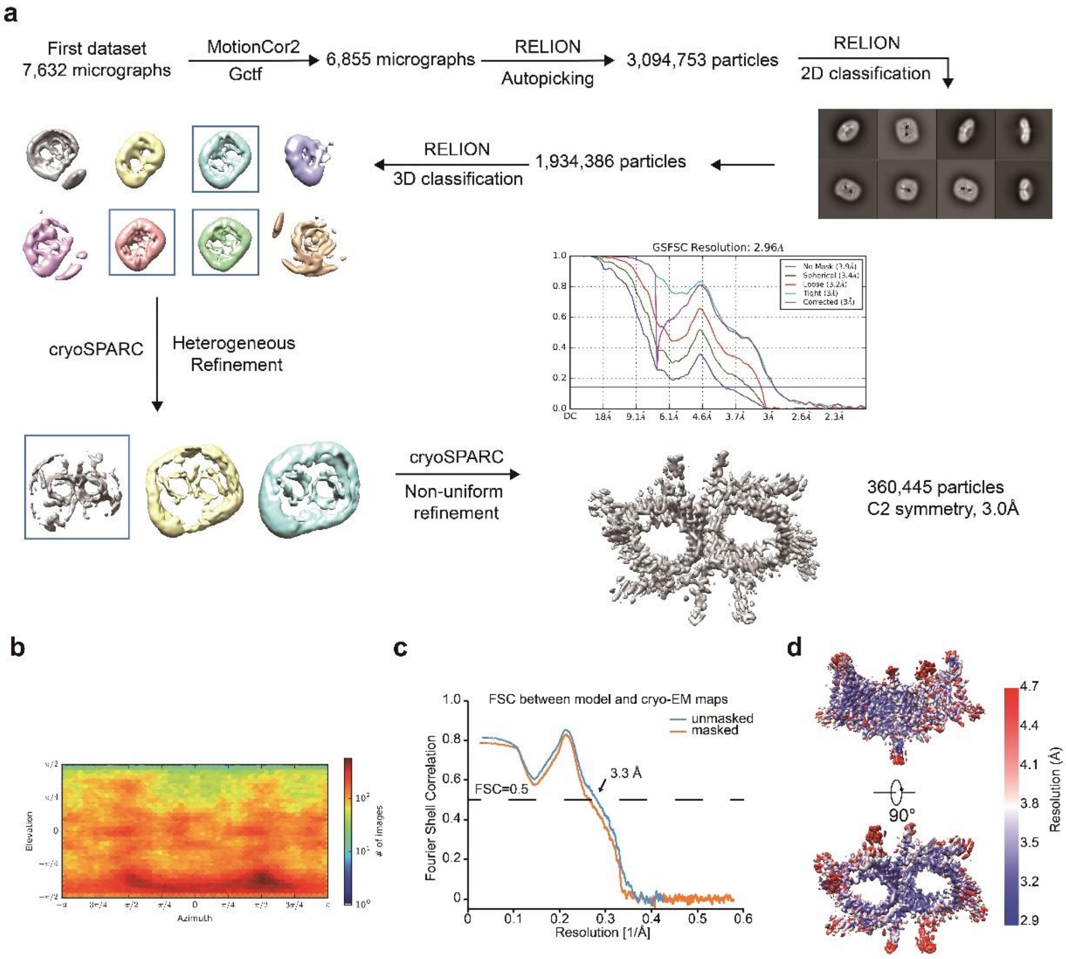

Supplementary Fig. S2 Cryo-EM reconstruction of the TOM core complex. **a** Single-particle cryo-EM data processing scheme using RELION and cryoSPARC. **b** Angular distribution of particles included in the final cryo-EM reconstruction for the TOM core complex. **c** FSC of the refined model versus the unmasked map (blue curve) and masked map (red curve) for the TOM core complex. **d** Cryo-EM density map for the TOM core complex, the different colors indicate the local resolution.

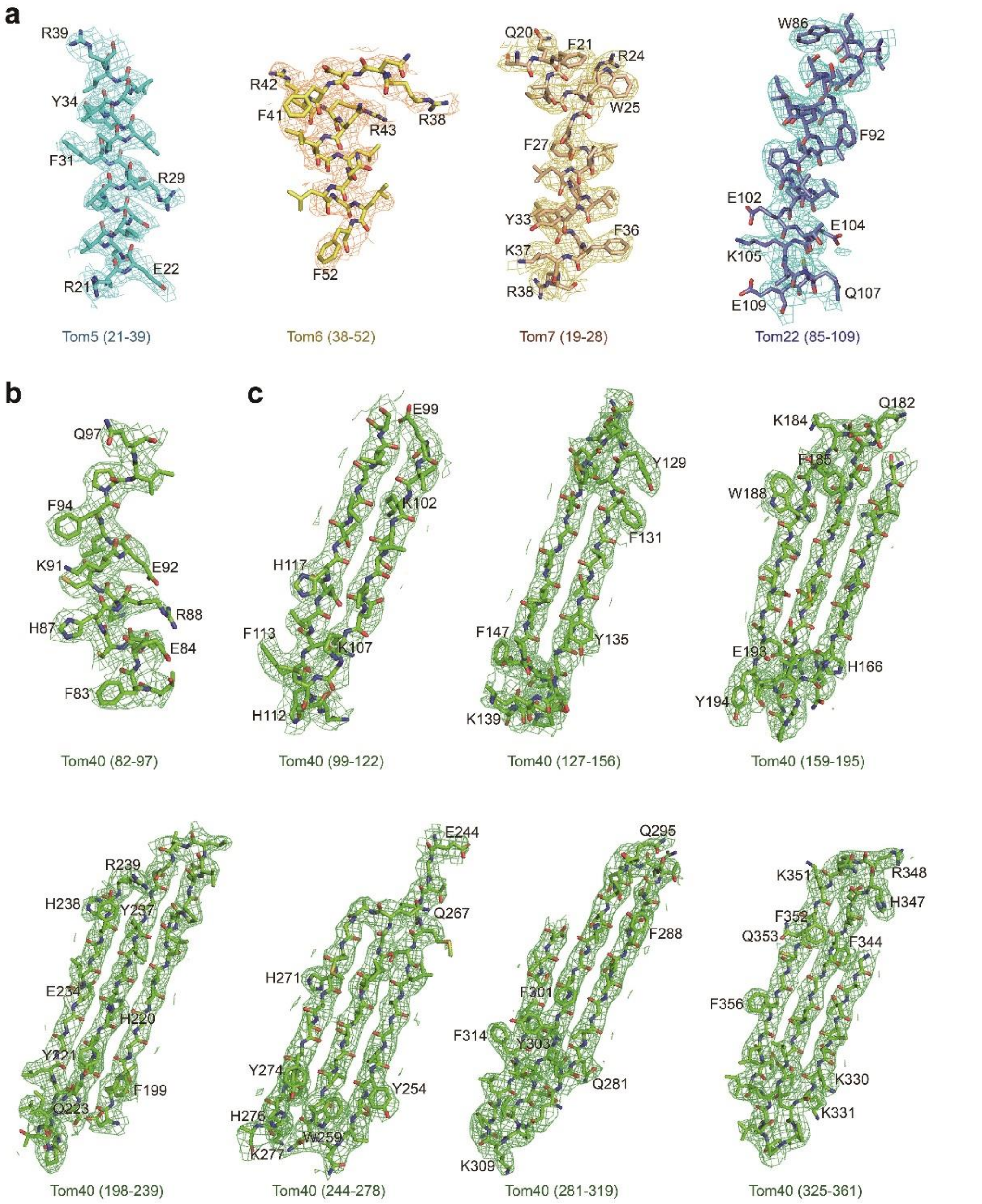

Supplementary Fig. S3 EM maps of representative segments of the TOM core complex. a-c Fragments of the TOM core complex with the corresponding cryo-EM densities shown as a mesh at 5σ. **a** Fragments of Tom5, Tom6, Tom7, Tom22. **b** N-terminal extended helix fragment of Tom40 **c** β-strand fragments of Tom40.

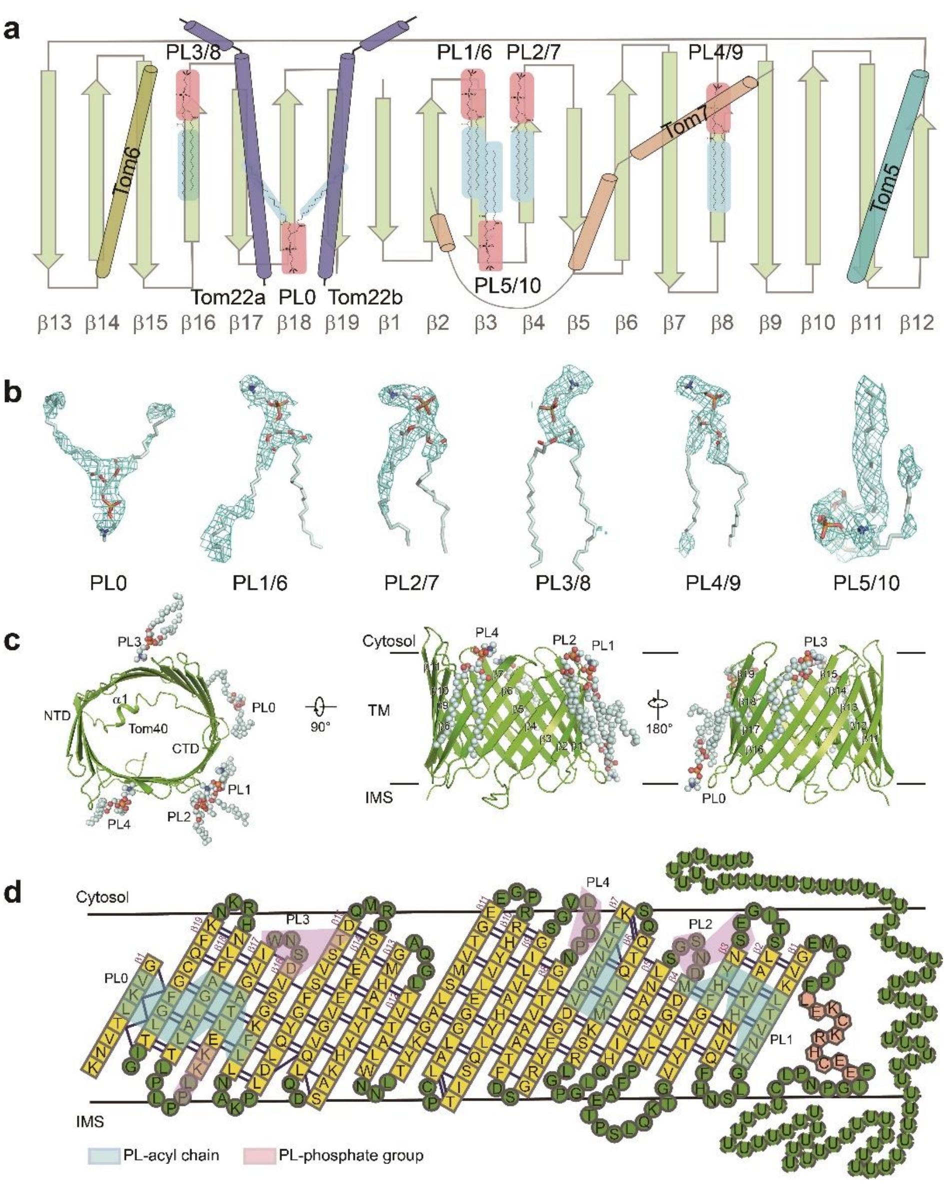

Supplementary Fig. S4 Topology of the human dimeric TOM core complex. **a** Topology diagram of the TOM core complex. The secondary elements and lipids are labeled. **b** Density maps of lipids (PL0 to PL10). **c** The lipids (PL0 to PL4) surrounding one of the Tom40 β-barrels are shown as spheres. **d** Location of the residues in Tom40. The residues bound to the head groups of lipids are indicated by pink shadows, and those bound to the acyl chains of lipids are indicated by cyan shadows.

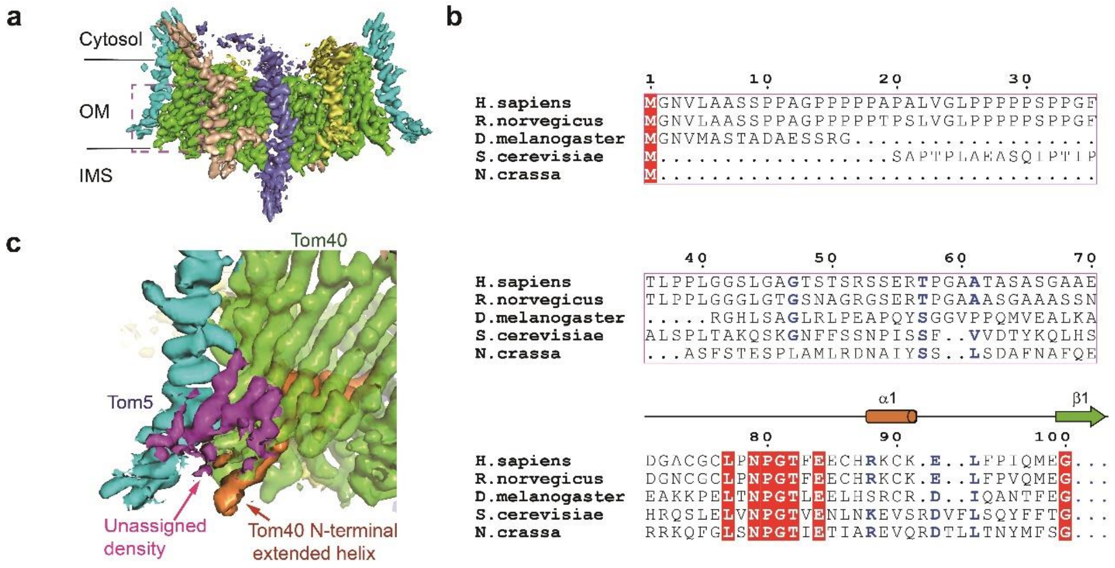

Supplementary Fig. S5 N-terminus of Tom40 exposed to the cytosol. a EM density map of the human TOM core complex. **b** Sequence alignment of the NTD in Tom40 among different species. **c** An unassigned density (shown in purple).

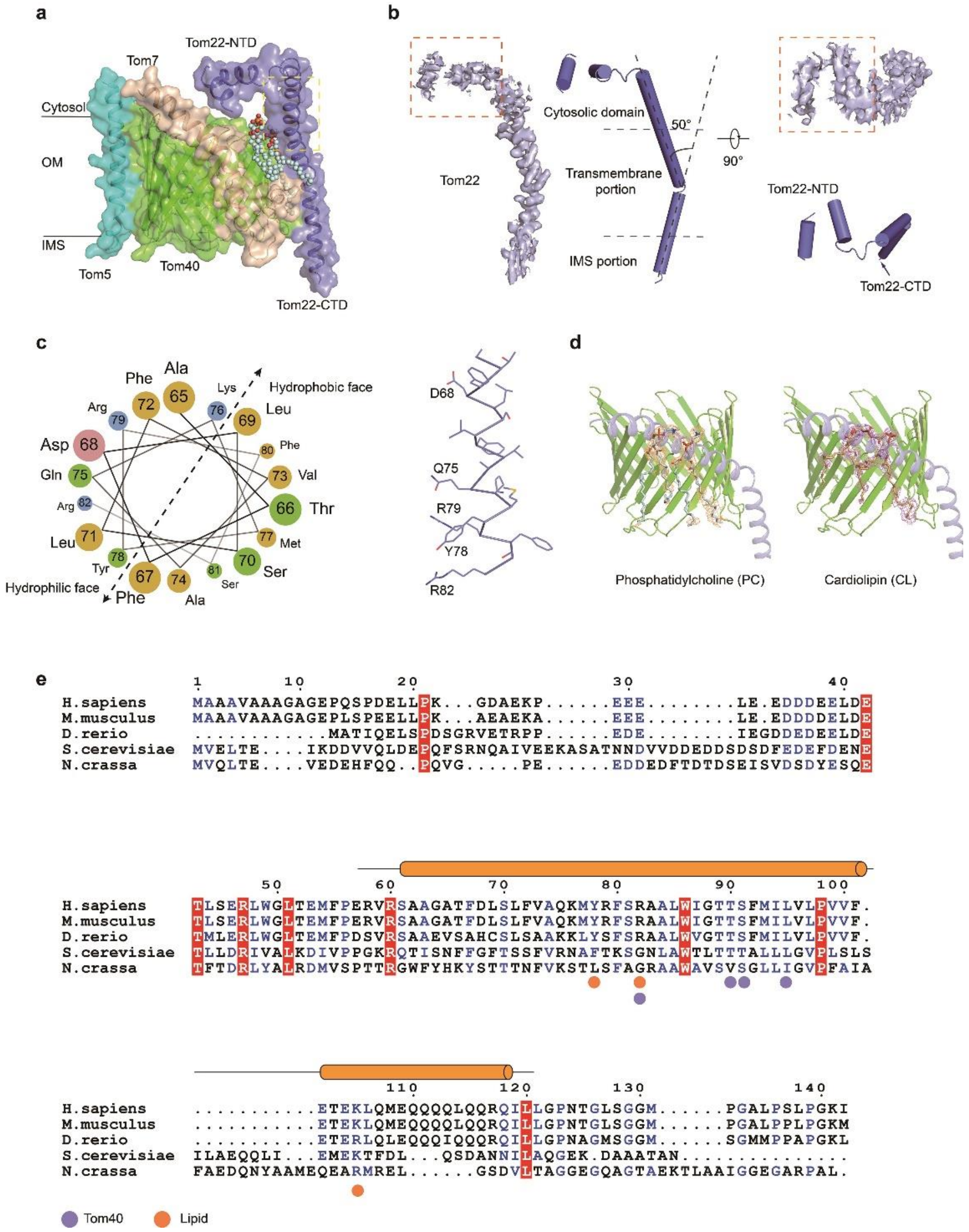

Supplementary Fig. S6 N-terminus of Tom22 plays a role in substrate binding. a Surface model of the TOM core complex. The NTD of Tom22 is indicated by a yellow dotted box. **b** Surface and cartoon model of Tom22. Tom22 consists of a cytosolic domain, a transmembrane portion, and an IMS portion. The NTD of Tom22 is highlighted with a brown dotted box. **c** Helical wheel indicating the residues in the NTD of Tom22. One side of the helix represents a hydrophobic face, and the other side represents a hydrophilic face. **d** The density map of lipids. Two phosphatidylcholines (PCs) (PL1 and PL2) are shown in orange (left panel). The cardiolipin (CL) molecules is shown in magenta (right panel). Previous studies have reported that mutation of the phospholipid transacylase tafazzin remodeling the fatty acyl side chains of cardiolipin causes severe human Barth syndrome. In yeast, CL is crucial for assembly of the TOM complex[20]. The key roles of PC and PE in stabilizing the yeast TOM complex have also been reported[21, 22]. **e** Sequence alignment of Tom22. Residues interact with lipids and Tom40 subunit are highlighted with brown and purple circles, respectively.

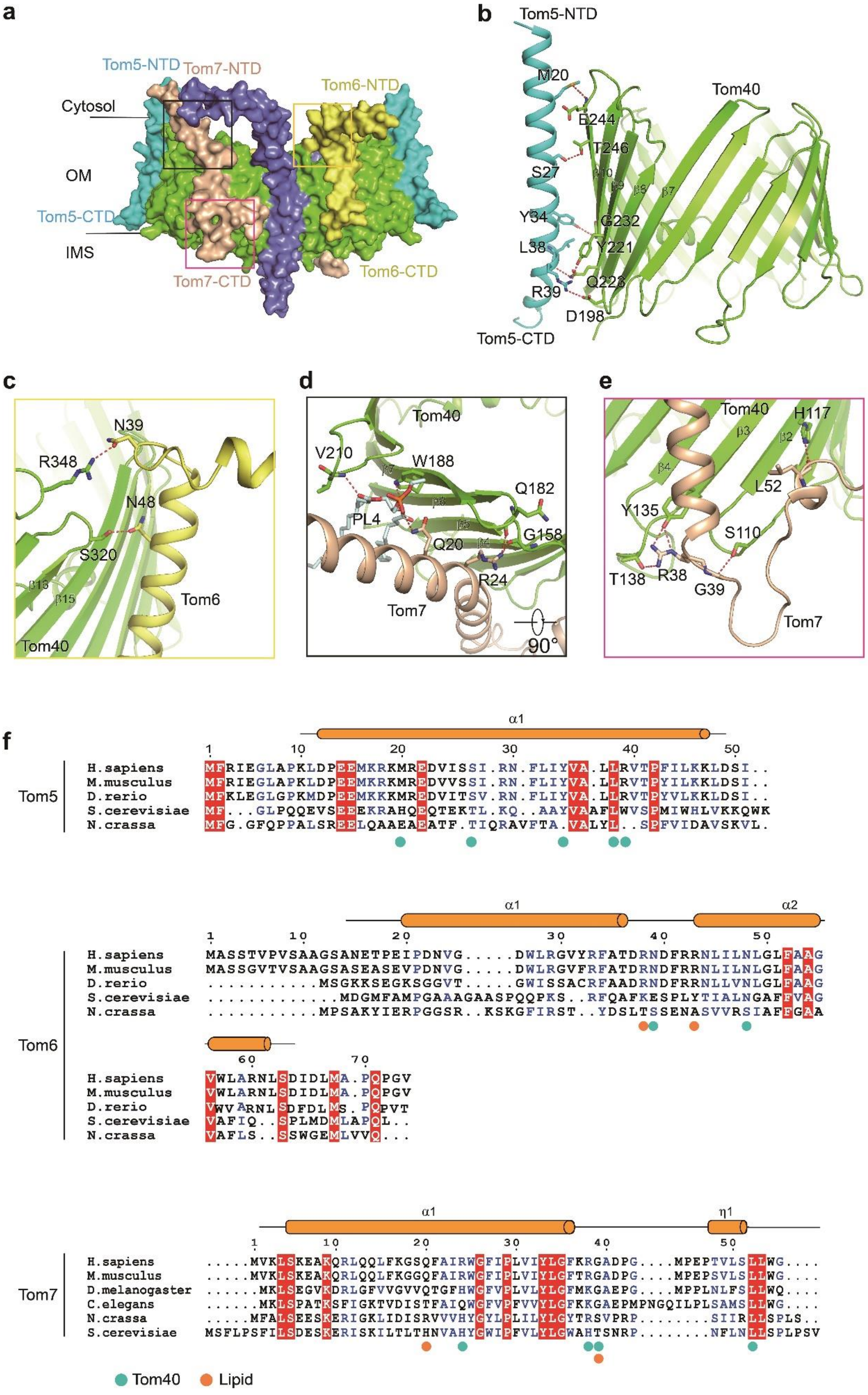
a
Tom5-NTD
Tom7-NTD
Tom6-NTD
Cytosol
OM
Tom5-CTD
IMS
Tom7-CTD
Tom6-CTD
b
Tom5-NTD
M20
E244
T246
S27
Tom40
Y34
G232
L38
Y221
Q228
R39
D198
Tom5-CTD
c
N39
R348
N48
S320
Tom6
Tom40
d
Tom40
V210
W188
Q182
PL4
Q20
G158
R24
Tom7
90°
e
Tom40
H117
L52
Y135
S110
T138
R38
G39
Tom7
f
Tom5
H.sapiens MFRIEGLAPKLDPEEMKRKMREDVISSI.RN.FLIYVA.LLRVTPFILKKLDSI..
M.musculus MFRIEGLAPKLDPEEMKRKMREDVVSSI.RN.FLIYVA.LLRVTPYILKKLDSI..
D.rerio MFKLEGLGPKMDPEEMKKKMREDVITSV.RN.FLIYVA.LLRVTPYVLKKLDSI..
S.cerevisiae MF...GLPQQEVSEEEKRAHQEQTEKTL.KQ..AAYVAAFLWVSPMIWHLVKKQWK
N.crassa MFG.GFQPPALSREELQAAEAEATF.TIQRAVFTA.VALYL..SPFVIDAVSKVL.
Tom6
H.sapiens MASSTVPVSAAGSANETPEIPDNVG.....DWLRGVYRFATDRNDFRRNLILNLGLFAAG
M.musculus MASSGVTVSAAGSASEASEVPDNVG.....DWLRGVFRFATDRNDFRRNLILNLGLFAAG
D.rerio ...........MSGKKSEGKSGGVT.....GWISSACRFAADRNDFRRNLLVNLGLFAAG
S.cerevisiae ............MDGMFAMPGAAAGAASPQQPKS..RFQAFKESPLYTIALNGAFFVAG
N.crassa ...........MPSAKYIERPGGSR..KSKGFIRST..YDSLTSSENASVVRSIAFFGAA
H.sapiens VWLARNLSDIDLMA.PQPGV
M.musculus VWLARNLSDIDLMA.PQPGV
D.rerio VWVARNLSDFDLMS.PQPVT
S.cerevisiae VAFIQ..SPLMDMLAPQL..
N.crassa VAFLS..SSWGEMLVVQ...
Tom7
H.sapiens .....MVKLSKEAKQRLQQLFKGSQFAIRWGFIPLVIYLGFKRGADPG....MPEPTVLSLLWG....
M.musculus .....MVKLSKEAKQRLQQLFKGGQFAIRWGFIPLVIYLGFTRGADPG....MPEPSVLSLLWG....
D.melanogaster ......MKLSEGVKDRLGFVVGVVQTGFHWGFVPLVLYLGFMKGAEPG....MPPLNLFSLLWQ....
C.elegans ......MKLSPATKSFIGKTVDISTFAIQWGFVPFVVYLGFKKGAEPMPNGQILPLSAMSLLWG....
N.crassa .....MFALSEESKERIGKLIDISRVVHYGYLPLILYLGYTRSVPRP........SIIRLLSPLS..
S.cerevisiae MSFLPSFILSDESKERISKILTLTHNVAHYGWIPFVLYLGWAHTSNRP........NFLNLLSPLPSV
Tom40
Lipid

Supplementary Fig. S7 Interactions between Tom40 and small Tom proteins. a Overall structure shown in surface. **b-e** Interactions between **b)** Tom40 and Tom5, **c)** Tom40 and Tom6, **d)** Tom40 and Tom7-NTD, and **e)** Tom40 and Tom7-CTD. The residues related to these interactions are indicated by sticks. **b** M20 and S27 of Tom5 interact with E244 and T246 of Tom40 near the cytosol, whereas Y34, L38 and R39 of Tom5 form hydrogen bond networks with Q223, G232, D198 and Y221 of Tom40 within the IMS. **c** N39 and N48 of Tom6 contact R348 and S320 of Tom40 by hydrogen bonds, respectively. **d** The side chain of R24 from the NTD of Tom7 forms hydrogen bonds with the main chains of G158 and Q182 of Tom40. PL4 contacts Tom7 (Q20) and Tom40 (W188, V210). **e** R38, G39, and L52 in the CTD of Tom7 recognize S110, Y135, T138, and H117 of Tom40 via hydrogen bonds. **f** Sequence alignments of Tom5, Tom6, and Tom7.

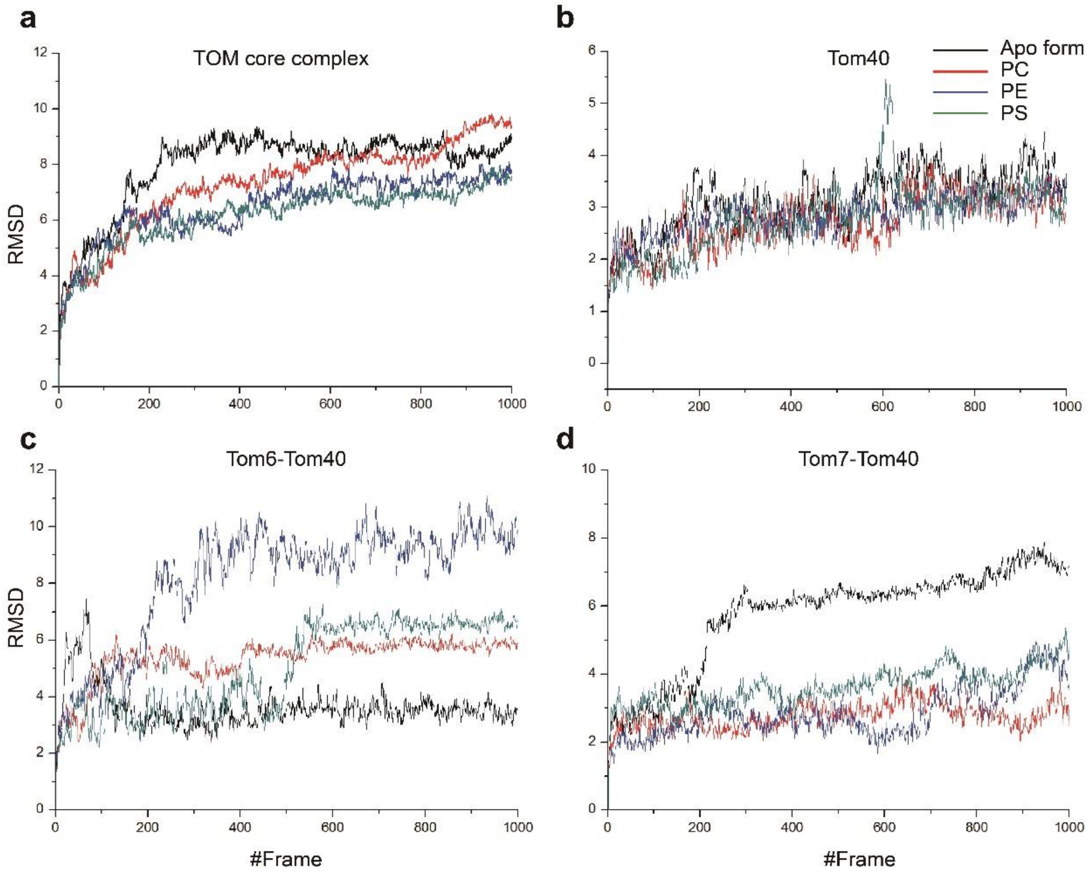

Supplementary Fig. S8 Lipids contribute to the stability of the TOM core complex. a Molecular dynamics simulation reveals that three lipids (PC, PE, and PS) improve the stability of the structure of TOM core complex with all 11 lipids. **b** These lipids do not improve the stability of the structure of Tom40 with PL0, PL1, PL2, PL3, and PL4. **c** PL3 disrupts the stability of the Tom40-Tom6 structure with PL3. **d** PL5 facilitates the stability of the Tom40-Tom7 structure with PL5.

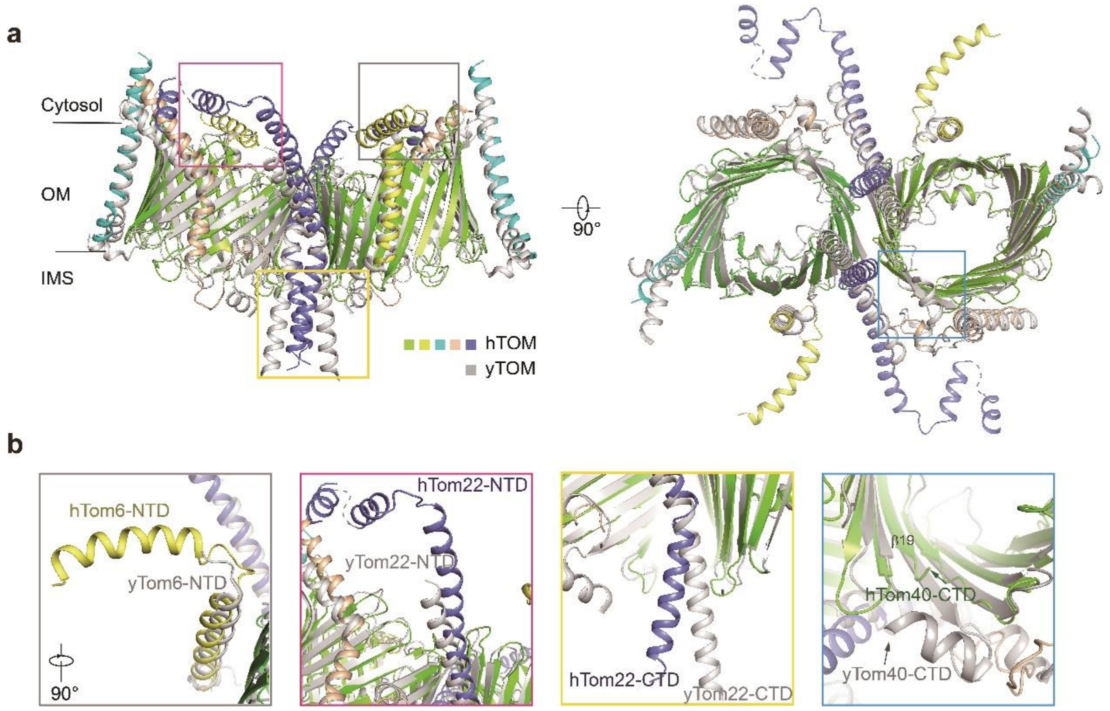

Supplementary Fig. S9 Structural alignment between the human TOM core complex and the yeast TOM core complex. a Side view (left panel); top view (right panel). **b** Comparisons among human Tom6, Tom22, and Tom40 and their yeast orthologs. The subunits of the yeast TOM core complex are shown in gray.

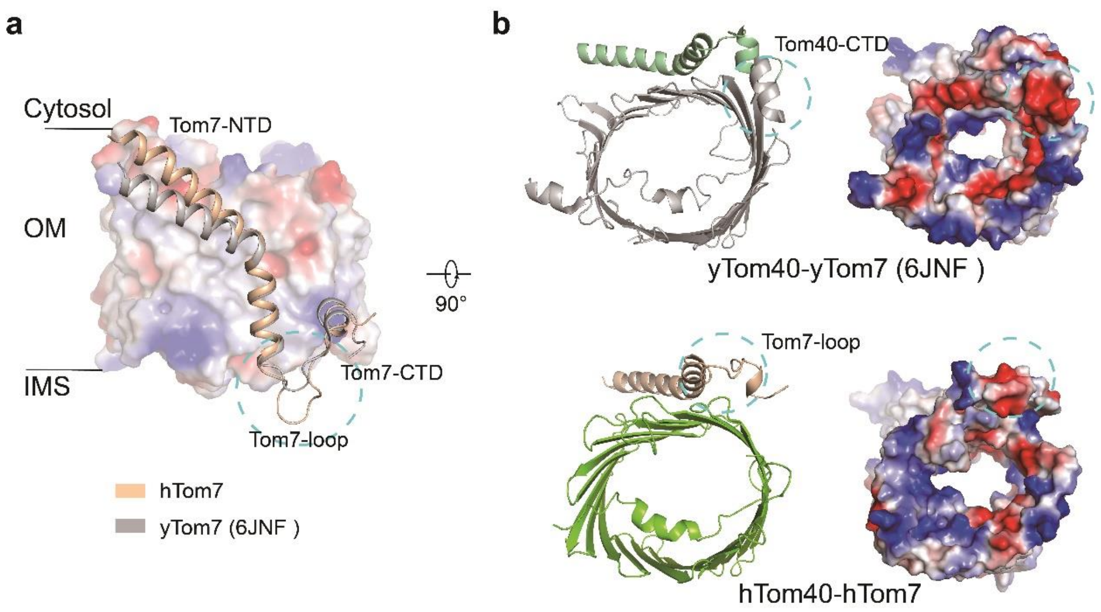

Supplementary Fig. S10 The human Tom7 connecting loop is related to precursor protein binding on the IMS side. **a** Structural alignment of human and yeast Tom7. The connecting loop is highlighted with a cyan dotted circle. **b** The substrate release patches of yTom40 and hTom40-Tom7 are indicated by cyan dotted circles.

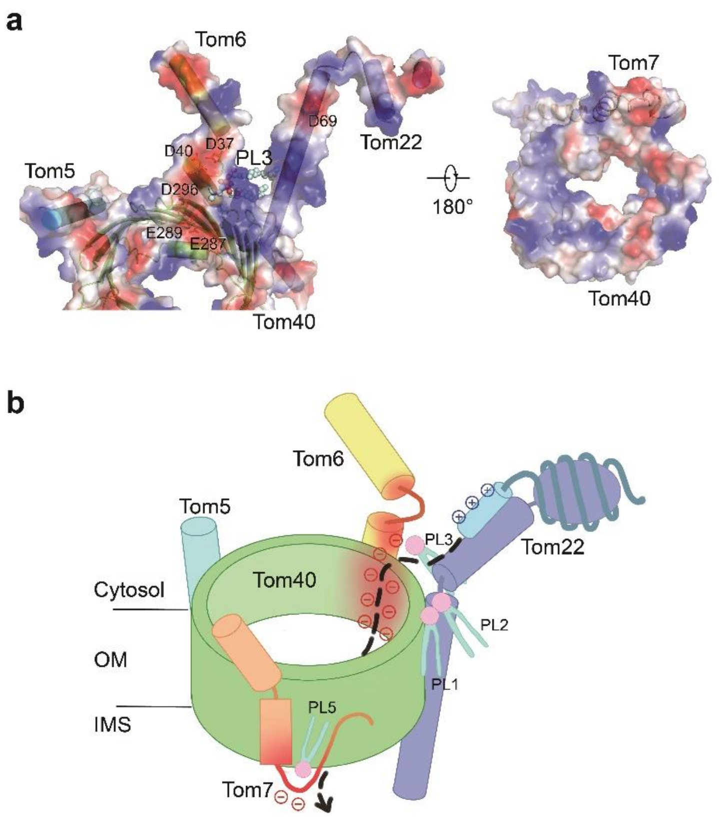

Supplementary Fig. S11 A proposed pathway of precursor proteins tranlocating through the TOM core complex. a Surface electrostatic potential of the TOM core complex from two different views. **b** Cartoon model of the presequence substrate (shown in slate) passing through the Tom40 channel. The possible pathway is indicated by black dotted arrows. The precursor protein is first recognized by the receptor, then received by the N-terminal cytosolic domain of Tom22, and subsequently presented to the negatively charged region of Tom6, where the precursor protein enters the Tom40 pore and continues to be translocated downwards.

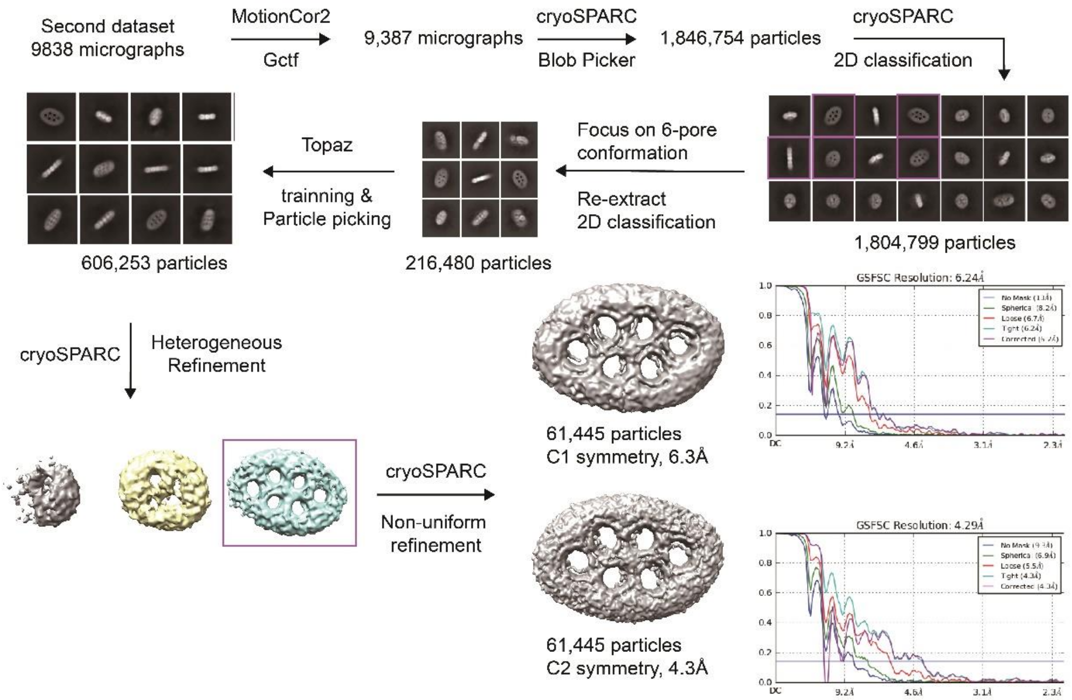

Supplementary Fig. S12 Cryo-EM reconstruction of trimeric TOM complex.

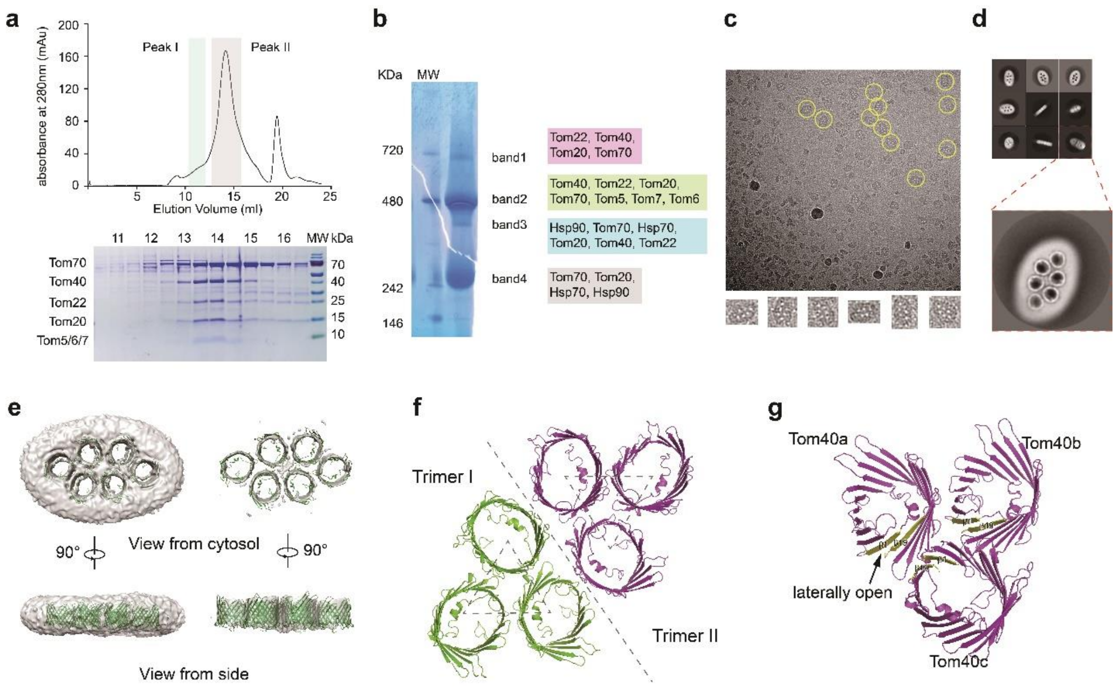

Supplementary Fig. S13 Structure of the human dimer of the trimeric TOM complex. **a** Representative gel filtration chromatography of the dimer of the trimeric TOM complex. Peak 1 was pooled for cryo-EM study. **b** BN-PAGE analysis of the cryo-EM sample from peak 1. Four bands were observed and analyzed by mass spectrometry. **c** Representative electron micrograph and 2D class averages of the dimer of the trimeric TOM complex. **d** 2D classification of the six pores. **e, f** Density and 3D model of the six pores. **g** Conformational change leading to exposure to the membranes of β1 and β19 in Tom40a.

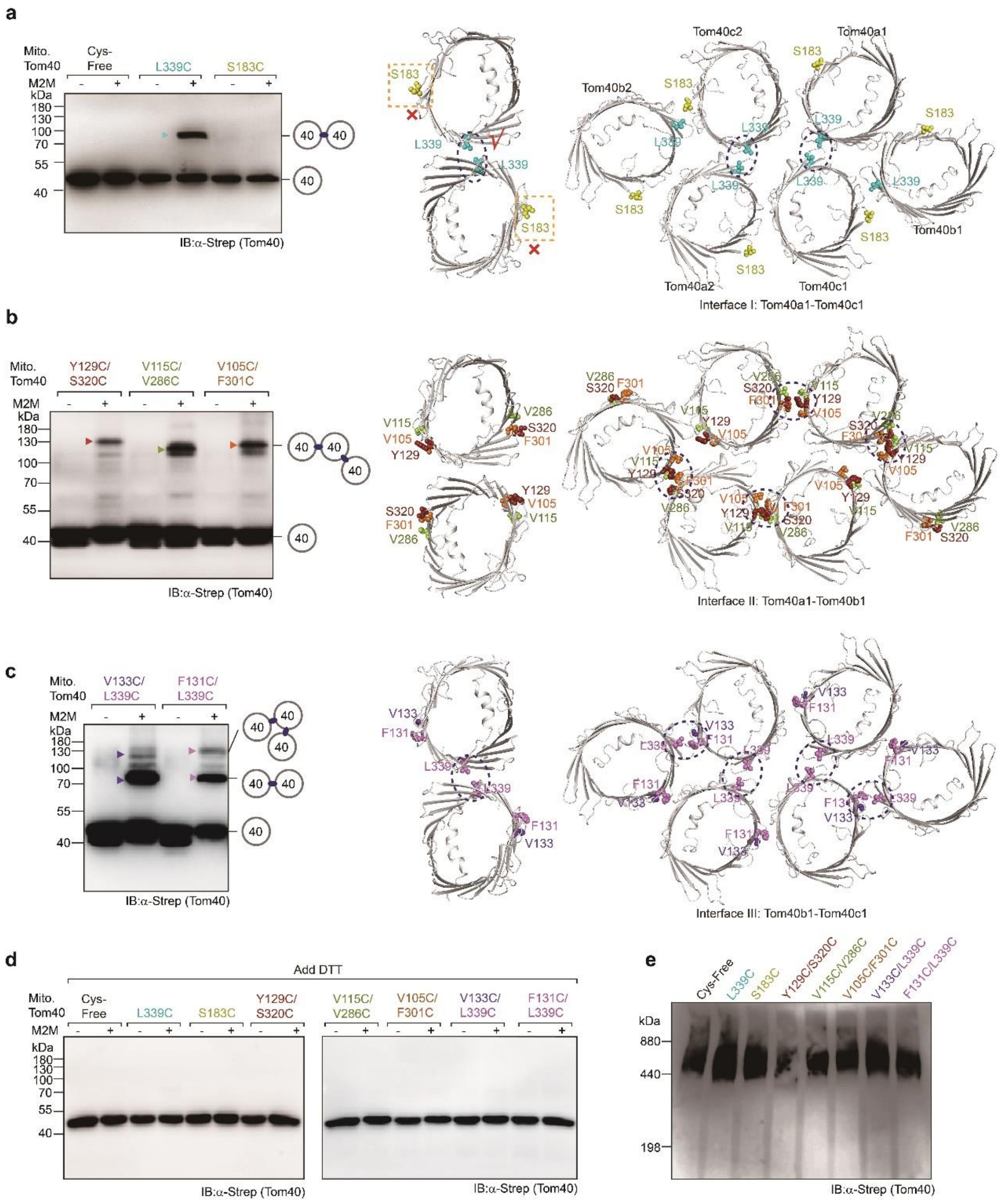

Supplementary Fig. S14 ***In organelle*** **crosslinking validation of the human trimeric TOM complex. a-c** Isolated mitochondria were treated with (+) or without (-) the crosslinker M2M and analyzed by SDS-PAGE and immunoblotting with antibodies of strep tag (left panel). Tom40 contained no or an indicated cysteine. The crosslinked products are indicated by colored triangles on the gel. The schematic model of the dimeric or dimer of trimeric Tom40 are shown in the right panel. Potential sites for cross-linking are indicated by dotted circles. **a** L339 (β18) was located at the interface of dimeric TOM and interface I of trimeric TOM. S183 (β7) was located far apart in the TOM dimer or trimer such that no cross-linked products were produced. **b** For the interface between Tom40a (β14-β15-β16) and Tom40b (β4-β3-β2), double mutations were generated. Y129 (β3) and S320 (β16), V115 (β2) and V286 (β14), and V105 (β1) and F301 (β15) were mutated in pairs. These mutants exhibited a band of ~130 kDa after incubation of the crosslinker because

these sites were also located at the interface of Tom40a1 and Tom40a2. **c** For the interface between Tonm40b (β19-β18-β17) and Tom40c (β2-β3-β4), a double cysteine mutation at positions V133 (β3)/L339 (β18) and F131 (β3)/L339 (β18) were introduced. The ~90-kDa and ~130-kDa bands were observed simultaneously because the crosslinking of $Tom40^{L339C}$ could yield an ~90-kDa band of dimeric TOM (refer to Fig. a). **d** The cross-linked products disappeared when the isolated mitochondria were treated with 50 mM DTT. e) BN-PAGE analysis of the TOM complex with the indicated Tom40 Cys residues. The results confirmed that the Tom40 variant could form the TOM complex as the WT.

Table S1 Statistics of Cryo-EM data collection and refinement of the human TOM complex.

	TOM core complex (EMD-30421) (PDB 7CP9)	Dimer of trimeric TOM complex (EMD-30422)
**Data collection**		
EM equipment	FEI Titan Krios	FEI Titan Krios
Voltage (kV)	300	300
Detector	Gatan K2	Gatan K2
Pixel size (Å)	1.014	1.091
Electron dose (e-/Å^2)	60	48
Defocus range (μm)	-1.8 ~ -2.5	-1.8 ~ -2.5
**Reconstruction**		
Software	RELION 3.0/ cryoSPARC	cryoSPARC
Number of used Particles	360,445	59,104
Symmetry imposed	C2	C2
FSC threshold	0.143	0.143
Final Resolution (Å)	3.0	4.3
**Model building**		
Software	COOT/ROSETTA	
**Refinement**		
Software	PHENIX	
Model resolution (Å)	3.3	
FSC threshold	0.5	
Average Fourier shell	0.827	
**Model composition**		
Protein residues	1,021	
Side chain assigned	1,003	
Lipid/Detergent	11	
**Validation**		
R.m.s deviations		
Bonds length (Å)	0.005	
Bonds Angle (˚)	0.692	
B factors (Å^2)		
Protein	67.89	
Lipid/Detergent	68.37	
**Ramachandran plot statistics (%)**		
Preferred	93.48	
Allowed	6.52	
Outlier	0.00	
Clash score	8.46	
Molprobity score	2.53	

Table S2 Summary of model building for the human TOM core complex.

	Length	Domain (copies)	PDB code	Modeling	Resolution (Å)	Chain ID
**Tom5**	51	11-48 (2)		de novo	3.2-4.2	A, B
**Tom6**	74	15-63 (2)		de novo	3.2-4.5	C, D
**Tom7**	55	4-55 (2)		de novo	3.0-3.8	E, F
**Tom22**	142	29-118 (2)		de novo	3.0-4.0	G, H
**Tom40**	361	76-361 (2)	5O8O	HM	2.9-3.5	I, J

Under the column labeled "Modeling", HM stands for homology modelling;

References

1 Lu PL, Bai XC, Ma D *et al.* Three-dimensional structure of human gamma-secretase. *Nature*. **512**:166-170 (2014).

2 Chari A, Haselbach D, Kirves JM *et al.* ProteoPlex: stability optimization of macromolecular complexes by sparse-matrix screening of chemical space. *Nat Methods*. **12**:859-865 (2015).

3 Wittig I, Braun HP, Schagger H. Blue native PAGE. *Nat Protoc*. **1**:418-428 (2006).

4 Mastronarde DN. Automated electron microscope tomography using robust prediction of specimen movements. *J Struct Biol.* **152**:36-51 (2005).

5 Zheng SQ, Palovcak E, Armache JP, Verba KA, Cheng YF, Agard DA. MotionCor2: anisotropic correction of beam-induced motion for improved cryo-electron microscopy. *Nat Methods*. **14**:331-332 (2017).

6 Zhang K. Gctf: Real-time CTF determination and correction. *J Struct Bio.* **193**:1-12 (2016).

7 Kimanius D, Forsberg BO, Scheres SHW, Lindahl E. Accelerated cryo-EM structure determination with parallelisation using GPUs in RELION-2. *Elife*.**5**, e18722 (2016).

8 Punjani A, Rubinstein JL, Fleet DJ, Brubaker MA. cryoSPARC: algorithms for rapid unsupervised cryo-EM structure determination. *Nat Methods*. **14**:290-296 (2017).

9 Chen SX, McMullan G, Faruqi AR *et al.* High-resolution noise substitution to measure overfitting and validate resolution in 3D structure determination by single particle electron cryomicroscopy. *Ultramicroscopy*. **135**:24-35 (2013).

10 Bausewein T, Mills DJ, Langer JD, Nitschke B, Nussberger S, Kuehlbrandt W. Cryo-EM Structure of the TOM Core Complex from *Neurospora crassa*. *Cell.* **170**:693-700 (2017).

11 Emsley P, Cowtan K. Coot: model-building tools for molecular graphics. *Acta Crystallogr D* Biol Crystallogr. **60**:2126-2132 (2004).

12 DiMaio F, Tyka MD, Baker ML, Chiu W, Baker D. Refinement of Protein Structures into Low-Resolution Density Maps Using Rosetta. *J Mol Biol.* **392**:181-190 (2009).

13 Song YF, DiMaio F, Wang RYR *et al.* High-Resolution Comparative Modeling with RosettaCM. *Structure*. **21**:1735-1742 (2013).

14 Dimaio F, Song Y, Li X *et al.* Atomic-accuracy models from 4.5- cryo-electron microscopy data with density-guided iterative local refinement. *Nat Methods*. **12**:361-365 (2015).

15 Adams PD, Afonine PV, Bunkoczi G *et al.* PHENIX: a comprehensive Python-based system for macromolecular structure solution. *Acta Crystallogr D Biol Crystallogr.* **66**:213-221 (2010).

16 Davis IW, Leaver-Fay A, Chen VB *et al.* MolProbity: all-atom contacts and structure validation for proteins and nucleic acids. *Nucleic Acids Res.* **35**:W375-W383 (2007).

17 Case. DA, Betz. RM, Cerutti. DS *et al.* AMBER 16, University of California, San Francisco. 2016.

18 Jakalian A, Jack DB, Bayly CI. Fast, efficient generation of high-quality atomic charges. AM1-BCC model: II. Parameterization and validation. *J Comput Chem* 2002; **23**:1623-1641.

19 Wang J, Wolf RM, Caldwell JW, Kollman PA, Case DA. Development and testing of a general amber force field. *J Comput Chem*; **25**:1157-1174 (2004).

20 Gebert N, Joshi AS, Kutik S *et al.* Mitochondrial Cardiolipin Involved in Outer-Membrane Protein Biogenesis: Implications for Barth Syndrome. *Curr Biol.* **19**:2133-2139 (2009).

21 Becker T, Horvath SE, Bottinger L, Gebert N, Daum G, Pfanner N. Role of Phosphatidylethanolamine in the Biogenesis of Mitochondrial Outer Membrane Proteins. *J Biol Chem*. **288**:16451-16459 (2013).

22 Schuler MH, Di Bartolomeo F, Bottinger L *et al.* Phosphatidylcholine Affects the Role of the Sorting and Assembly Machinery in the Biogenesis of Mitochondrial beta-Barrel Proteins. *J Biol Chem.* **290**:26523-26532 (2015).
